# Supplementary material for: Inverse proximity effect in semiconductor Majorana nanowires
Source: Beilstein J Nanotechnol. 2018 Apr 16;9:1184–93. doi: 10.3762/bjnano.9.109 (PMC5942367; doi:10.3762/bjnano.9.109)
Supplement: File 1 — Derivation of the model Equation 6 and Equation 7. [file Beilstein_J_Nanotechnol-09-1184-s001.pdf]

Supporting Information

for

Inverse proximity effect in semiconductor Majorana nanowires

Alexander A. Kopasov*[1], Ivan M. Khaymovich[1,2] and Alexander S. Mel'nikov[1,3]

Address: [1]Institute for Physics of Microstructures, Russian Academy of Sciences, 603950 Nizhny Novgorod, GSP-105, Russia; [2]Max Planck Institute for the Physics of Complex Systems, 01187 Dresden, Germany and [3]Lobachevsky State University of Nizhny Novgorod, 23 Gagarina, 603950 Nizhny Novgorod, Russia

Email: Alexander A. Kopasov - kopasov@ipmras.ru

* Corresponding author

Derivation of the model equations

Here we present the derivation of the model Equations 6–7. The Hamiltonian of the system (Equation 1) given in the main text consists of three terms (Equations 2–4) describing the superconducting shell, the semiconducting nanowire, and the tunneling terms between these subsystems, respectively. The notations are also given in the main text.

The field operators in the shell are normalized in the following way:

$$\left[\psi_\sigma(\mathbf{r}), \psi^\dagger_{\sigma'}(\mathbf{r}')\right]_+ = \frac{1}{d_s R_w}\delta_{\sigma\sigma'}\delta(\varphi-\varphi')\delta(y-y'). \tag{S1}$$

Here $[A,B]_+ = AB + BA$ denotes the anticommutator of two operators A and B. The operators in the nanowire satisfy the following anticommutation relations:

$$\left[a_\sigma(y), a^\dagger_{\sigma'}(y')\right]_+ = \frac{1}{S_w}\delta_{\sigma\sigma}\delta(y-y')\ . \tag{S2}$$

Now we proceed with the definitions of the Matsubara Green's functions in the shell. We adopt the following spinor notation in the Nambu space:

$$\Psi(\mathbf{x}) = \begin{pmatrix} \psi_\uparrow(\mathbf{x}) \\ \psi_\downarrow(\mathbf{x}) \end{pmatrix} \text{ and } \widetilde{\Psi}(\mathbf{x}) = \begin{pmatrix} \psi_\uparrow(\mathbf{x}) & \psi_\downarrow(\mathbf{x}) \end{pmatrix}\ . \tag{S3}$$

Here $\mathbf{x} = (\mathbf{r}, \tau)$, while τ is the imaginary time variable in the standard Matsubara technique. Using the above notations, we introduce the Green's functions as follows:

$$\check{G}_s = \left\langle T_\tau \left[\begin{pmatrix} -\Psi(\mathbf{x}_1) \\ \widetilde{\Psi}^\dagger(\mathbf{x}_1) \end{pmatrix} \otimes \begin{pmatrix} \Psi^\dagger(\mathbf{x}_2) & -\widetilde{\Psi}(\mathbf{x}_2) \end{pmatrix} \right] \right\rangle = \begin{pmatrix} \hat{G}_s(\mathbf{x}_1,\mathbf{x}_2) & \hat{F}_s(\mathbf{x}_1,\mathbf{x}_2) \\ \hat{F}^\dagger_s(\mathbf{x}_1,\mathbf{x}_2) & \hat{\bar{G}}_s(\mathbf{x}_1,\mathbf{x}_2) \end{pmatrix}, \tag{S4}$$

where $\langle ... \rangle$ denotes the Gibbs statistical average and T_τ is the time-ordering operator. The definition of the Green's functions of the wire is given by Equation S3 and Equation S4 with the replacement

of the field operators $\psi_\alpha(\mathbf{x}) \to a_\alpha(\mathbf{y})$ along with the replacement of the subscripts $s \to w$, where $\mathbf{y} = (y, \tau)$.

Using the Nambu spinor notation presented in the above equation, we introduce the mixed Green's functions in the spin-Nambu space:

$$\check{G}_t = \left\langle T_\tau \left[\begin{pmatrix} -\Psi(\mathbf{x}_1) \\ \widetilde{\Psi}^\dagger(\mathbf{x}_1) \end{pmatrix} \otimes \begin{pmatrix} a^\dagger(\mathbf{y}_2) & -\widetilde{a}(\mathbf{y}_2) \end{pmatrix} \right] \right\rangle = \begin{pmatrix} \hat{G}_t(\mathbf{x}_1,\mathbf{y}_2) & \hat{F}_t(\mathbf{x}_1,\mathbf{y}_2) \\ \hat{F}_t^\dagger(\mathbf{x}_1,\mathbf{y}_2) & \hat{\overline{G}}_t(\mathbf{x}_1,\mathbf{y}_2) \end{pmatrix} . \tag{S5}$$

We start the derivation of Gor'kov equations (Equation 10) by writing the equations for the mixed Matsubara Green's functions (Equation S5) in the frequency–coordinate representation:

$$[\check{G}_s^{(0)}(\mathbf{r}_1)]^{-1}\check{G}_t(\mathbf{r}_1,y_2) - (1/\zeta)\check{\mathscr{T}}(\mathbf{r}_1)\check{G}_w(y_1,y_2) = 0 , \tag{S6}$$

$$\check{G}_t(\mathbf{r}_1,y_2)[\check{G}_w^{(0)}(y_2)]^{-1} - \zeta \left\langle \check{G}_s(\mathbf{r}_1,\mathbf{r}_2)\check{\mathscr{T}}(\mathbf{r}_2) \right\rangle_{\varphi_2} = 0 , \tag{S7}$$

where

$$[\check{G}_s^{(0)}(\mathbf{r})]^{-1} = i\omega_n - \varepsilon_s(\mathbf{r})\check{\tau}_z + \check{\Delta} , \tag{S8}$$

$$[\check{G}_w^{(0)}(y)]^{-1} = i\omega_n - \varepsilon_w(y) + i\alpha\partial_y\hat{\sigma}_x - h\hat{\sigma}_y , \tag{S9}$$

$\zeta = \sqrt{d_s/\pi R_w}$, $\check{\mathscr{T}}(\mathbf{r}) = [\mathscr{T}(\mathbf{r})\check{\Pi}_{z_+} - \mathscr{T}^\dagger(\mathbf{r})\check{\Pi}_{z_-}]$, $\mathscr{T}(\mathbf{r})$ is the tunneling amplitude, $\check{\Pi}_{z_\pm} = (1 \pm \check{\tau}_z)/2$, and $\langle ... \rangle_\varphi = \int d\varphi$. The solution of Equation S6 with the boundary conditions corresponding to the isolated superconductor takes the form

$$\check{G}_t(\mathbf{r}_1,y_2) = \sqrt{d_s R_w S_w}\langle \check{G}_s^{(0)}(\mathbf{r}_1,\mathbf{r})\check{\mathscr{T}}(\mathbf{r})\check{G}_w(y,y_2)\rangle_{\mathbf{r}} , \tag{S10}$$

where $\langle ... \rangle_{\mathbf{r}} = \int dy\, d\varphi$. The Green's function of the isolated superconductor satisfies the following equation:

$$[\check{G}_s^{(0)}(\mathbf{r}_1)]^{-1}\check{G}_s^{(0)}(\mathbf{r}_1,\mathbf{r}_2) = \check{1}(d_s R_w)^{-1}\delta(\varphi_1 - \varphi_2)\delta(y_1 - y_2) . \tag{S11}$$

Substituting Equation S10 into the equation for the Green's function in the wire

$$\check{G}_w^{-1}(y_1)\check{G}_w(y_1,y_2) - \zeta\langle\check{\mathscr{T}}^\dagger(\mathbf{r}_1)\check{G}_t(\mathbf{r}_1,y_2)\rangle_{\varphi_1} = \check{1}S_w^{-1}\delta(y_1-y_2) \tag{S12}$$

and performing the ensemble averaging over the random tunneling amplitudes

$$\overline{\mathscr{T}(\mathbf{r})\mathscr{T}(\mathbf{r}')} = t^2\ell_c\delta(y-y')\delta(\varphi-\varphi')\ , \tag{S13}$$

we get Dyson-Gor'kov equations for the Green's functions of the wire

$$\left[\check{G}_w^{-1}(y_1) - \check{\Sigma}_w(y_1)\right]\check{G}_w(y_1,y_2) = \check{1}S_w^{-1}\delta(y_1-y_2)\ , \tag{S14}$$

with the self-energy part taken in the limit of an isolated superconducting shell

$$\check{\Sigma}_w(y) = d_sR_wt^2\ell_c\check{\tau}_z\langle\check{G}_s^{(0)}(\mathbf{r},\mathbf{r})\rangle_\varphi\check{\tau}_z\ . \tag{S15}$$

The Dyson-Gor'kov equations in the superconducting shell are derived using the same arguments as for the previous case

$$\left[\check{G}_s^{-1}(\mathbf{r}_1) - \check{\Sigma}_s(\mathbf{r}_1)\right]\check{G}_s^{(0)}(\mathbf{r}_1,\mathbf{r}_2) = \check{1}(d_sR_w)^{-1}\delta(\varphi_1-\varphi_2)\delta(y_1-y_2)\ , \tag{S16}$$

with the self-energy part taken in the limit of an isolated semiconducting wire

$$\check{\Sigma}_s(\mathbf{r}) = S_wt^2\ell_c\check{\tau}_z\check{G}_w^{(0)}(y,y)\check{\tau}_z\ , \tag{S17}$$

the solution of Equation S7, and with the Green's function of the isolated wire satisfying the following equation

$$\check{G}_w^{-1}(y_1)\check{G}_w^{(0)}(y_1,y_2) = \check{1}S_w^{-1}\delta(y_1-y_2)\ . \tag{S18}$$

To take exact boundary conditions into account we follow the procedure suggested in [1] replacing $\check{G}_s^{(0)}(\mathbf{r}_1, \mathbf{r}_2)$ and $\check{G}_w^{(0)}(y_1, y_2)$ in Equation S15 and Equation S17 with the exact ones

$$\check{\Sigma}_w(y) = d_s R_w t^2 \ell_c \check{\tau}_z \langle \check{G}_s(\mathbf{r}, \mathbf{r}) \rangle_\varphi \check{\tau}_z \,, \tag{S19}$$

$$\check{\Sigma}_s(\mathbf{r}) = S_w t^2 \ell_c \check{\tau}_z \check{G}_w(y, y) \check{\tau}_z \,. \tag{S20}$$

Finally the Fourier transform of Equation S14 and Equation S16 with the self-energies (Equation S19 and Equation S20) along with the renormalization of the Green's functions $\check{G}_w \to \check{G}_w / S_w$ and $\check{G}_s \to \check{G}_s / d_s$ completes the derivation of Equations 6–7 in the main text.

References

1. McMillan, W. L. *Phys. Rev.* **1968**, *175*, 537–542. doi:10.1103/PhysRev.175.537.
